# Apigenin protects ischemic stroke by regulating intestinal microbiota homeostasis, regulates brain metabolic profile

**DOI:** 10.3389/fphar.2025.1553081

**Published:** 2025-03-07

**Authors:** Jinjian Li, Qiaoli Xu, Xiaoming Xu, Wei He, Hui Zhang, Haoxu Ren, Yue Wang, Xu Wang, Dexi Zhao

**Affiliations:** ^1^ Department of Encephalopathy, Hospital of Changchun University of Chinese Medicine, Changchun, Jilin, China; ^2^ Department of Encephalopathy, Changchun Hospital of Traditional Chinese Medicine, Changchun, Jilin, China; ^3^ College of Traditional Chinese Medicine, Changchun University of Chinese Medicine, Changchun, Jilin, China

**Keywords:** apigenin, ischemic stroke, ATP, intestinal flora, AMPK

## Abstract

**Background and Objective:**

Ischemic stroke is a cerebrovascular disease with highly incidence. Previous research has demonstrated that apigenin provides protective effects against ischemic stroke. However, it remains unclear whether apigenin can regulate intestinal flora against ischemic stroke.

**Methods:**

In this study, we evaluated the regulatory effects of apigenin on intestinal microbiota using a middle cerebral artery occlusion rat model. The protective impact of apigenin on brain damage in ischemic stroke rats was assessed through Nissl staining, hematoxylin and eosin staining, and immunohistochemistry. Additionally, we employed 16S rRNA sequencing to analyze intestinal contents and utilized non-targeted metabolomics to investigate the effects of apigenin on brain metabolites, thereby exploring its mechanism of action. AMPK levels were detected by Western blot and immunohistochemistry. The kit was used to detect oxidative stress and inflammation.

**Results:**

The intervention with apigenin resulted in significant alterations in the intestinal flora, characterized by an increase in the abundance of probiotic species and a decrease in harmful flora, alongside notable changes in brain metabolite profiles. This protective effect is attributed to apigenin’s promotion of AMPK expression and enhancement of energy metabolism in the context of ischemic stroke. In addition, apigenin improved oxidative stress and inflammation in ischemic stroke.

**Conclusion:**

These findings suggest that apigenin exerts a protective effect on ischemic stroke through the AMPK signaling pathway by modulating intestinal flora and associated metabolites. Consequently, apigenin emerges as a therapeutic candidate warranting further investigation.

## 1 Introduction

Stroke is a leading cause of death and disability, affecting nearly 100 million people worldwide (Carcel et al., 2020). Ischemic stroke (IS) accounts for over 80% of cases among these patients (Virani et al., 2020). Recombinant tissue plasminogen activator (rtPA) is the only treatment approved by the US Food and Drug Administration for IS; however, its use is restricted by strict time windows, potential side effects, and contraindications (Hacke et al., 2008; Macrez et al., 2011). Therefore, it is essential to identify safe and effective medications for the prevention and treatment of IS, aiming to mitigate the damage caused by IS and minimize associated complications (Wang et al., 2024a).

Intestinal flora, comprising a diverse array of microorganisms residing in the gastrointestinal tract, play a significant role in regulating various physiological functions of the host (Li et al., 2022). The bidirectional regulation theory of the gut-brain axis posits that intestinal flora are integral to cerebrovascular and neurological diseases, influencing brain function and contributing to the maintenance of brain health through multiple mechanisms (Wang and Wang, 2016). Changes in intestinal microorganisms and microbiota metabolites are closely associated with intestinal function, thrombosis, and IS (Zhu et al., 2016; Tan et al., 2020). Consequently, modifying intestinal microbial metabolites and enhancing intestinal barrier function represent valuable strategies for the treatment and prevention of IS in the context of cardiovascular and cerebrovascular diseases.

Apigenin (APG), a natural polyphenol, is affordable, easy to extract and widely distributed in fruits and vegetables such as parsley, Chinese celery and chamomile, etc. Moreover, APG can regulate the human gut microbiota (Wang et al., 2019). APG has the ability to regulate the composition of intestinal microorganisms in the context of tumor diseases. It promotes the proliferation of beneficial bacteria while inhibiting the growth of harmful bacteria, thereby enhancing the intestinal environment and mitigating inflammation and oxidative stress (Bian et al., 2020). Our previous studies found that APG treated IS by anti-oxidative stress and protecting the blood-brain barrier (Wang X. et al., 2022; Ping et al., 2024; Wang et al., 2024b). However, the regulatory effect of APG on intestinal microbiome and its effects on brain metabolites in IS remain unclear.

Metabolomics is an emerging technology that employs various modern analytical techniques to measure the dynamic changes of small molecule metabolites in biological organisms, thereby characterizing and elucidating the status of life activities (Li et al., 2024). This approach offers a more accurate and direct reflection of the terminal and phenotypic information of biological systems, providing a novel perspective for understanding the multifactorial mechanisms of diseases and for comprehensively evaluating drug effects.

This study utilized 16S rRNA full-length sequencing and liquid chromatography-mass spectrometry (LC-MS) to perform a non-targeted metabolomic analysis of brain tissue metabolites and intestinal microorganisms in MCAO rats treated with APG. The objective was to explore the regulatory effects of APG on intestinal microorganisms and their influence on brain metabolism in the context of IS. This research provides a scientific foundation for the application of APG in the treatment of IS.

## 2 Material and methods

### 2.1 Reagents

APG was acquired from Meryer Chemical Technology Co., Ltd., located in Shanghai. The ATP, ATPase, malondialdehyde (MDA) assay kit, total superoxide dismutase (SOD) assay kit, glutathione (GSH), Interleukin-1beta (IL1β),and Interleukin-6(IL6) assay kits were sourced from Nanjing Jiancheng Bioengineering Institute. A nylon wire featuring a rounded tip was obtained from BEIJING XINONTECH CO.

### 2.2 Animals and experimental design

60 adult male Sprague-Dawley (SD) rats weighing approximately 250 ± 30 g were acquired from Liaoning Changsheng Biotechnology Co., Ltd., (License No. SCXK LIAO 2020-0001). These rats were accommodated in the Animal Experiment Center at the School of Public Health, Jilin University, China (Experimental unit License number: SYXK JI 2018-0001). All procedures involving the animals adhered to the guidelines set forth by the National Institutes of Health for the Care and Use of Laboratory Animals, as well as the standards established by the National Research Council of the National Academies. The rats were maintained under controlled environmental conditions, with an ambient temperature of approximately 23°C ± 1°C, a light/dark cycle of 12 h each, unrestricted access to food and water, and a humidity level maintained between 60% and 70%. This study received approval and was monitored by the Animal Experimentation Committee at Changchun University of Traditional Chinese Medicine (ID: 2023482).

The 60 rats were randomly divided into 4 groups, including the sham operation group (Control group), the model group (MCAO group), the APG group (60 mg/kg, APG group), the treat group (60 mg/kg, MCAO + APG group). The treatment group rat was treated with APG through gavage for 7 days consecutively, while the control and model groups received equivalent volumes of sterile saline. The dosage of APG was determined based on previous study (Ping et al., 2024). The treatment group received APG via gavage for a continuous duration of 7 days after MCAO model, while the control and model groups were administered identical volumes of sterile saline. Throughout the procedure, all animals had unrestricted access to food and water in a suitable environment. The rats were weighed and then anesthetized using an intraperitoneal injection of sodium pentobarbital (2%, 2 mL/kg), after which they were positioned in a ventral recumbent state, supported by a warming pad to ensure their core temperature remained around 37.0°C during the operation. The MCAO model was established utilizing Longa’s technique. An incision was made along the midline of the neck to reveal the common carotid artery (CCA), external carotid artery (ECA), and internal carotid artery (ICA) (Ping et al., 2024). Both the CCA and ECA were ligated, and a round-tipped nylon wire was delicately inserted into the ICA until slight resistance was encountered. The rats were euthanized 7 days afterward. The control group underwent identical surgical exposure procedures to the model group, excluding the insertion of nylon wires.

The neurological function of rats in each group was assessed using the Longa method 7 days after the pMCAO model was established. The criteria for scoring included the following: 0 points indicated normal neurological function; 1 point was assigned to rats that could not fully extend their contralateral forepaw; 2 points were given to rats that walked in a circular motion toward the hemiplegic side; 3 points indicated that rats tended to lie toward the hemiplegic side while at rest; and 4 points were assigned to rats that lost consciousness and were incapable of independent movement.

### 2.3 16S rDNA gene amplification and sequencing

Gensky Biotechnologies Inc., based in Shanghai, China, conducted sequencing of 16S rRNA amplicons. To extract DNA from the samples, they utilized the MagAttract PowerSoil DNA Isolation kit, following the manufacturer’s guidelines (Qiagen, Hilden, Germany). The amplification and sequencing of the V3-V4 variable region of the bacterial 16S rDNA gene was performed using the Illumina MiSeq platform, located in San Diego, CA, United States.

### 2.4 Gut microbiota analysis

Carefully measure the correct quantity of the sample into a 2 mL EP tube, then incorporate 600 µL of methanol (which includes 2-chloro-L-phenylalanine at 4 ppm and has been stored at −20°C), and vortex for 30 s. The subsequent analysis of the sample was carried out using the following procedure. Introduce steel balls, position in a tissue grinder, and perform grinding at a frequency of 50 Hz for 120 s. Sonicate the mixture for 10 min at ambient temperature, followed by centrifugation at 12,000 rpm for 10 min at 4°C. Filter the supernatant through a 0.22 μm membrane, and transfer the filtered solution into assay vials; 20 µL from each sample to be analyzed is amalgamated to create the QC samples (QC: employed to adjust for the deviations in mixed samples as well as errors stemming from the analyzing instrument; QC sampling is conducted if the total number of experimental samples exceeds 10; if there are 10 or fewer samples, QC sampling will not take place). The remainder of the samples will be utilized for LC-MS analysis. The complete metabolic analysis process involves processing the raw files through spectral mapping and searching databases, resulting in the identification and quantification of metabolites. Subsequently, a quality control assessment was performed to verify the accuracy and reliability of the results. Metabolites were further examined using multivariate statistical analysis to uncover differences between various groups. Correlation analyses among metabolites and hierarchical clustering were employed to ascertain the relationships between the samples and the metabolites. Ultimately, the metabolites were examined through metabolic pathways and additional functional assessments to elucidate the biological relevance of metabolite-associated biological significance.

### 2.5 Nissl’s staining

In summary, prepare fresh rat brain tissue by fixing it in a 10% formalin solution. Sections of paraffin should be cut to a thickness of 5 µm. Subsequently, dewax the paraffin sections and rehydrate them using graded alcohol solutions. Finally, the sections are dewaxed in xylene for 45 min and dehydrated through a gradient concentration of ethanol. The sections are then immersed in Nissl staining reagent for staining purposes.

### 2.6 Pathological and immunohistochemical detection of brain tissue

Hematoxylin and eosin (HE) staining was conducted for histological assessment. In summary, brain samples from each group were obtained and preserved in 10% paraformaldehyde. The tissue sections underwent dehydration, were processed using an automated tissue processor, and were then enveloped in paraffin. Sections of 4 µm thickness were created. HE staining followed after the sections were dewaxed and hydrated. Scanning of the sections was performed using a scanner.

Immunohistochemical staining was carried out on brain tissue sections that were fixed in paraformaldehyde and embedded in paraffin. Sections, measuring 3–4 μm in thickness, were treated with anti-Phospho Adenosine 5′-monophosphate (AMP)-activated protein kinase (p-AMPK) antibody. The primary antibody was allowed to incubate at 37°C for 1 hour. Following this, the brain slices received horseradish peroxidase-conjugated secondary antibody for 30 min at 37°C, and the color reaction was developed with diaminobenzidine as the chromogenic substrate. The final sections were counterstained with hematoxylin, dehydrated through graded ethanol and xylene, and images of the ischemic semi-dark bands were captured using a light microscope. The integral optical density values of positive cells were evaluated with ImageJ software.

### 2.7 Microbiological data analysis

The analysis of the sequence was conducted utilizing quantitative insights into microbial ecology (QIIME). Evaluating the differences within the microbial community and the impact of each differentially abundant taxon was achieved through linear discriminant analysis (LDA) effect size (LEfSe) with standard parameters (LDA score >2.0 and P < 0.05), aiding in the identification of biomarkers. Cluster analysis was carried out based on Bray-Curtis dissimilarity. Different groups were represented by distinct colors, and branch lengths indicated the distance between samples, with closer distances denoting similar compositions. The β-diversity of the microbial community was illustrated through principal coordinate analysis (PCoA) of weighted UniFrac distances, followed by ANOSIM analysis. The relative abundance of bacterial communities at both phylum and genus levels across the three groups was evaluated using the Kruskal–Wallis rank-sun test. The distribution of functional pathway genes was anticipated by predicting microbial functions through the phylogenetic investigation of communities via reconstruction of unobserved states 2 (PICRUSt2), utilizing high-quality sequences from KEGG (https://www.genome.ad.jp/).

### 2.8 Metabolomics data analysis

Orthogonal partial least squares discrimination analysis (OPLS-DA) served as the statistical tool for analyzing metabolic alterations between the two groups. The model’s validity was evaluated using the R^2^ and Q^2^ parameters, which indicated the interpretability and predictability of the model, effectively mitigating the risk of overfitting. In the OPLS-DA framework, the significance of the projection variables (VIP) was computed to assess the influence strength and explanatory capacity of the expression patterns of each metabolite on the classification and differentiation of the sample groups, aiding in the identification of marker metabolites. P values were determined through paired Student’s T-test, which was utilized for one-dimensional statistical analyses. Metabolites with VIP values greater than 1 and P values below 0.05 were deemed statistically significant. The differential metabolites were compiled and linked to biochemical pathways via metabolic enrichment analysis and pathway exploration using KEGG. The interrelationships between the different metabolites were determined using the Spearman rank correlation coefficient and visualized with a heatmap created in R software utilizing the “heatmap” package.

### 2.9 Western blot analysis

Total protein was extracted utilizing the Radioimmunoprecipitation assay buffer (RIPA) solution. The protein sample concentrations were measured using a BCA kit. A total of 30 µg of these protein samples were subjected to separation via sodium dodecyl sulfate–polyacrylamide gel electrophoresis (SDS-PAGE) and subsequently transferred onto a polyvinylidene difluoride (PVDF) membrane. Following this, the membranes were blocked with 5% BSA at room temperature for 60 min and then incubated overnight at 4°C with various antibodies: anti-p-AMPK (1:1,000, CST), and anti-GAPDH (1:10,000, CST). Afterward, the membranes were rinsed with TBST and incubated with a horseradish peroxidase-conjugated secondary antibody for 60 min. The blots were then visualized using ECL reagent (GE Healthcare, Piscataway, NJ, United States), and ImageJ software was employed to assess the band densities.

### 2.10 Oxidative stress and inflammation were detected by elisa

Brain tissue samples were obtained from the ischemic semi-dark zone for testing. Kits were used to detect MDA, GSH, SOD, IL1β, and IL6, following the manufacturer’s guidelines. In summary, a 10% tissue homogenate was initially created, the protein concentration was measured, and subsequent experiments were conducted in accordance with the provided instructions.

### 2.11 Statistical analysis

Data were analyzed using GraphPad Prism software. The findings are expressed as mean ± standard deviation (SD), and a one-way analysis of variance (ANOVA) was carried out, followed by Tukey’s *post hoc* analysis. Statistical significance was assessed at *P* < 0.05.

## 3 Results

### 3.1 1APG has protective effect on IS

**FIGURE 1:**
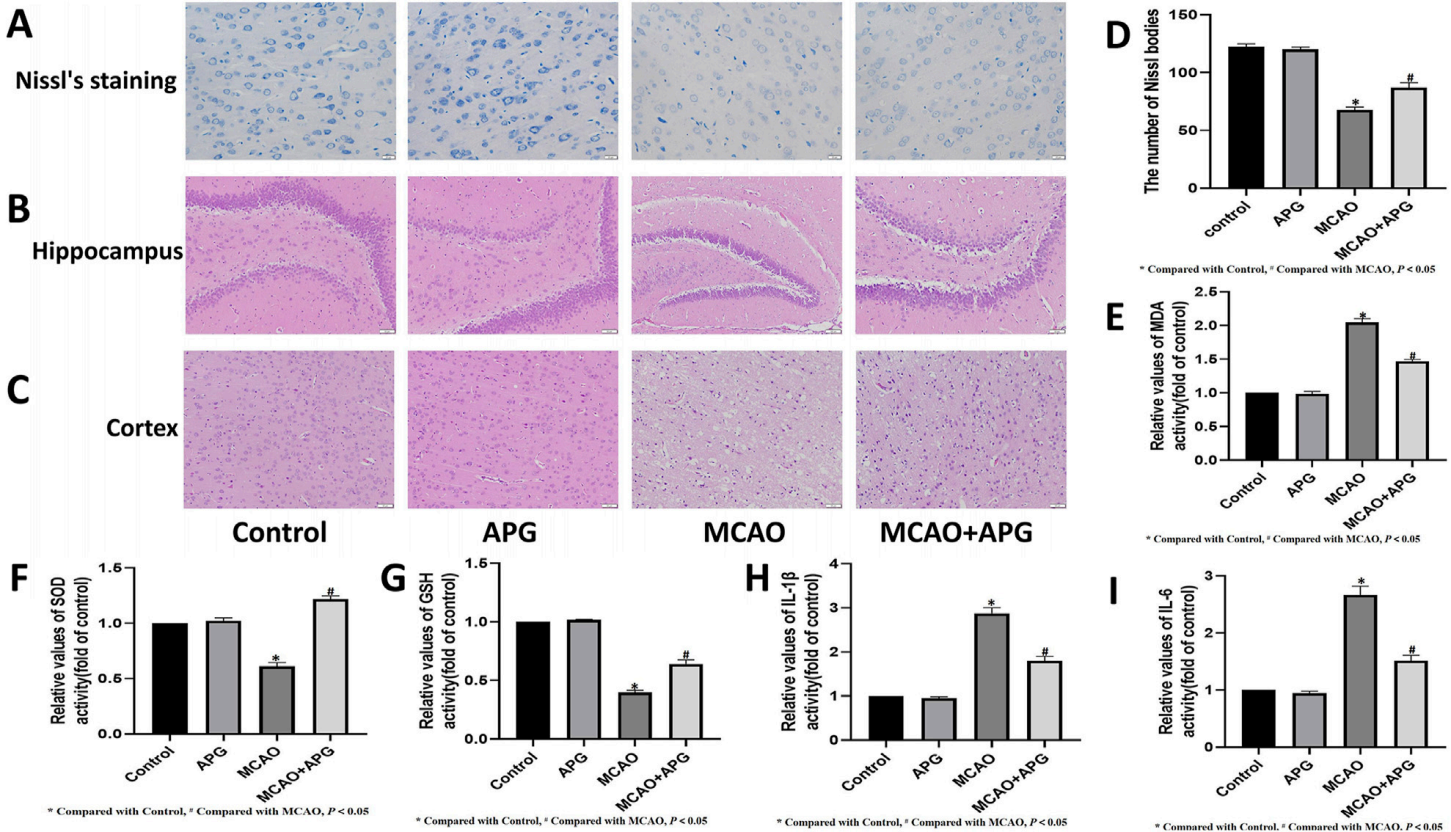
APG on pathological changes after MCAO. **(A)** Representative nissl’s staining images of the ischemic penumbra. Bar = 20 µm. **(B, C)**: Representative pathological images of the ischemic penumbra (Hippocampus and cortex). Bar = 50 µm. **(D)** The number of Nissl bodies in the cerebral cortex area neurons showed the extent of damage. **(E–I)** Statistical graph of MDA, SOD, GSH, IL1β, and IL6 (n = 3). *P < 0.05, compared with control; ^#^ *P* < 0.05, compared with MCAO.

The results revealed that the cortical structure was clearly visible in both the control group and the rats administered APG, characterized by large, round nuclei and a greater number of blue Nissl bodies within the cell layer. On the other hand, the MCAO model group displayed disorganized nerve fibers, with nuclei appearing either triangular or spindle-like, and a reduction or complete absence of Nissl bodies, which resulted in a notable decrease in their numbers. Nevertheless, after APG treatment, the Nissl bodies in the MCAO model rats were found to have been rejuvenated, and their quantity showed an increase (Figure 1A). And APG increased Nissl staining-positive cells in the penumbra tissue (Figure 1D), and promoted therecovery of neurological function (Figure 6C).

HE staining demonstrated edema, structural vacuolization, nuclear condensation, and a rise in eosinophils in the ischemic semi-dark band of rats from the MCAO group, with significant improvement noted after treatment with APG (Figures 1B, C).

### 3.2 16s rRNA sequencing revealed the changes of microflora after MCAO

**FIGURE 2:**
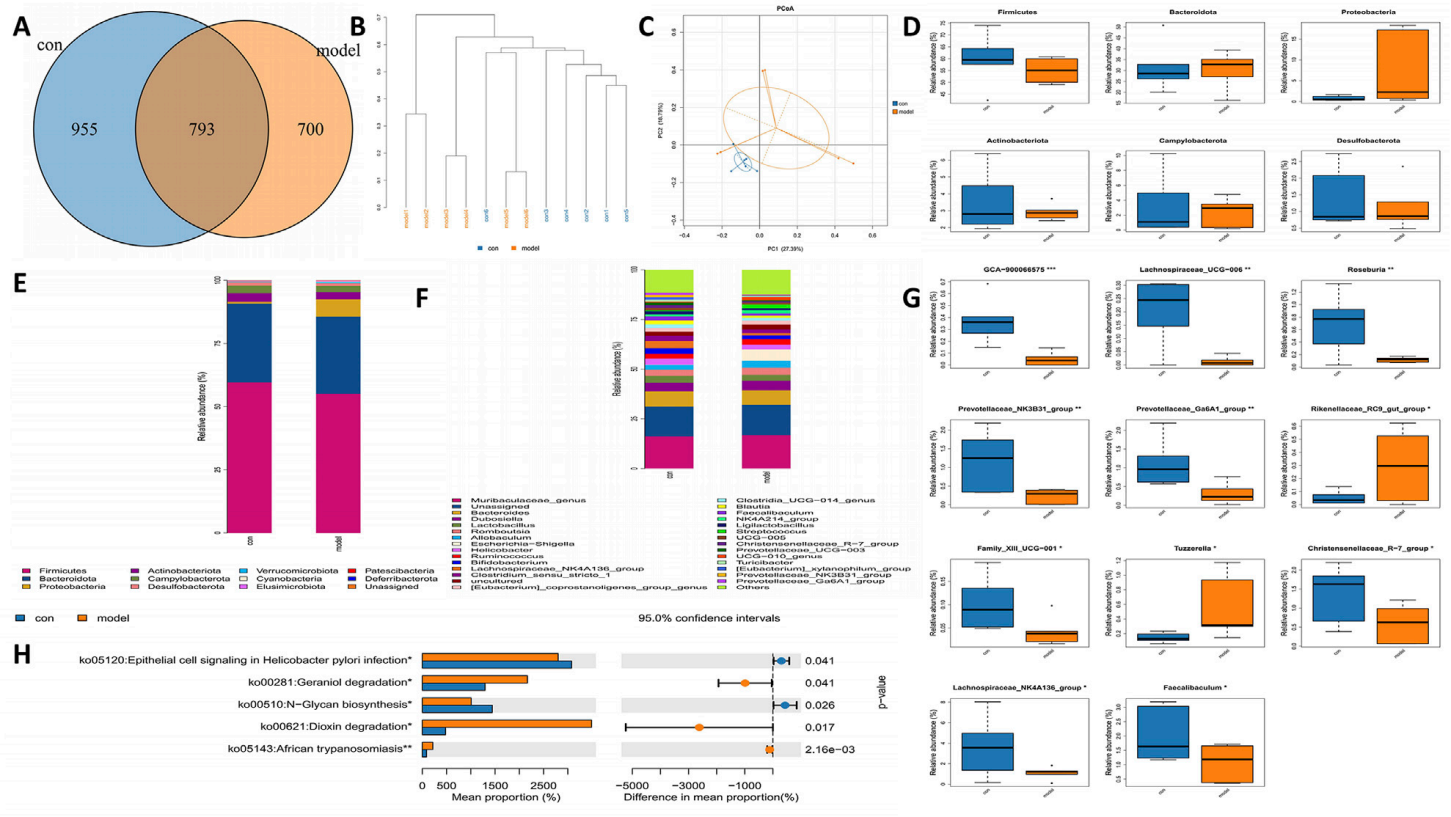
Alterations in the gut microbiota of MCAO rats. **(A)** Venn diagram of different gut microbiota between model and control groups. **(B)** Hierarchical clustering tree. Samples from different groups are colored differently, and the length of branches represents the distance between samples. The closer distance of sample indicates similar composition. **(C)** The bacterial community’s PCoA. The closer the distance, the greater the likeness. **(D)** Bacterial communities with statistically significant differences in abundance at the phylum level. **(E)** The distribution of gut microbiota relative abundance at the phylum level. **(F)** The distribution of gut microbiota relative abundance at the genus level. **(G)** Bacterial communities with statistically significant differences in abundance at the genus level. **(H)** Based on 16 s rRNA sequencing data, PICRUSt2 analysis was used to predict the differential metabolic pathways. *p < 0.05, **p < 0.01, ***p < 0.001.

Disruption of microbiota impacts the progression of disease. To further clarify the role of gut microbes in IS, we examined the gut microbiota in rats subjected to MCAO. Rat feces were collected for 16S rRNA sequencing, and we analyzed the Operational Taxonomic Units (OTUs) present in both control and model groups. The control group exhibited 955 OTUs, while the model group contained 700 OTUs, with 793 OTUs shared between the two groups (Figure 2A). Cluster analysis indicated a significant disparity in fecal species composition between the model group and the control group rats (Figure 2B). Principal Coordinate Analysis (PCoA) serves as a beta diversity analysis technique utilizing unifrac distance (Zhou et al., 2024). Each point depicted in the figure corresponds to a sample, with the varied colors representing the subgroups to which these samples are associated. A closer distribution of the points suggests a higher similarity among the samples. The findings revealed that the differences within the groups were not substantial, indicating a high level of similarity among the samples. There was no overlap observed between the two groups, and the separation along with the variability between groups was pronounced, indicating that the gut microbiota in the model group of rats underwent significant changes (Figure 2C). Additionally, a noteworthy reduction in the levels of Firmicutes, Bacteroidota, and Proteobacteria was identified in the model group (Figure 2D). Multi-sample species composition histograms are frequently employed as visual analysis tools to illustrate the species makeup present in each sample. We examined the microbiota composition structure at both the phylum and genus levels, selecting species with a relative abundance of over 1% for each sample for inclusion in the histograms. The results highlighted significant differences in the fecal microbiota composition between the control and model rats. At the phylum level, decreases in Firmicutes, Bacteroidetes, and Actinobacteriota were noted in the model group. Notably, there was a significant increase in Proteobacteria in the model group (Figure 2E). At the genus level, Lachnospiraceae showed a marked reduction in the model group, while *Escherichia*−*Shigella* exhibited a significant increase in this group (Figure 2F). Other genera also displayed significant differences between the two groups (Figure 2G). To predict functional changes in the intestinal microbiota of APG rats, we employed PICRUSt2 analysis. Predictions based on the KEGG database indicated that: signaling related to epithelial cells in *Helicobacter pylori* infection, geraniol degradation, N−glycan biosynthesis, and dioxin degradation were significantly elevated in the model group (*P* < 0.05) (Figure 2H).

### 3.3 16s rRNA sequencing revealed the changes of microflora after MCAO treatment with APG

**FIGURE 3:**
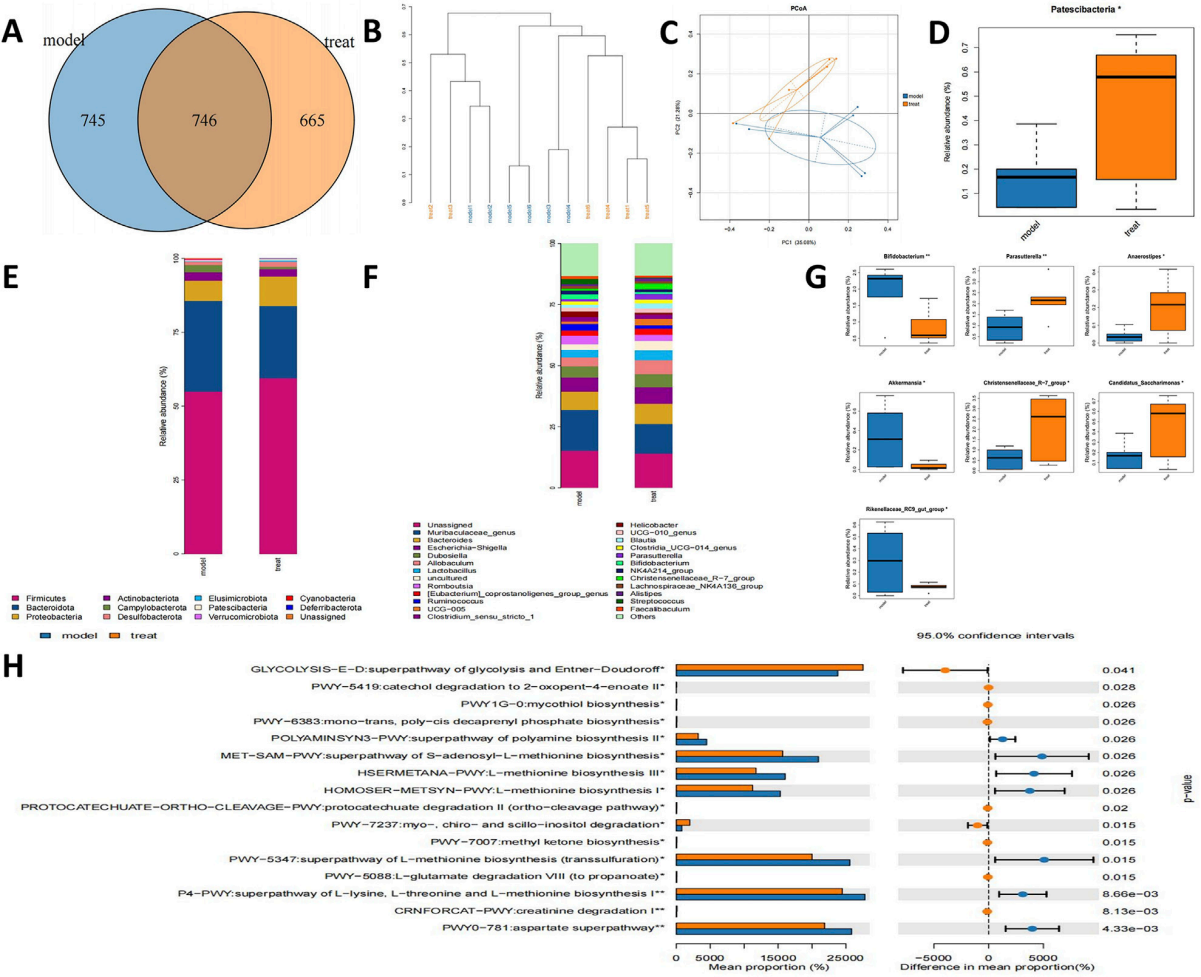
APG ameliorates gut microbiota in MCAO rats. **(A)** Venn diagram of different gut microbiota between model and treat groups. **(B)** The tree of hierarchical clustering. Different sets of samples are colored differently, and the length of the branches shows the distance between samples. The closer proximity of the samples implies a comparable makeup. **(C)** The bacterial community’s PCoA. The closer the distance, the greater the likeness. **(D)** Bacterial communities with statistically significant differences in abundance at the phylum level. **(E)** The distribution of gut microbiota relative abundance at the phylum level. **(F)** The distribution of gut microbiota relative abundance at the genus level. **(G)** Bacterial communities with statistically significant differences in abundance at the genus level. **(H)** Based on 16s rRNA sequencing data, PICRUSt2 analysis was used to predict the differential metabolic pathways. *p < 0.05, **p < 0.01, ***p < 0.001.

To explore the regulatory impacts of APG on the gut microbiome of MCAO rats, we collected fecal samples from various rat groups and conducted 16S rRNA sequencing. The APG and model groups exhibited 664 and 745 OUTs, respectively, resulting in a total of 746 OUTs across both groups (Figure 3A). Cluster analysis indicated a notable difference in the fecal species composition between the MCAO and APG groups (Figure 3B). Further, PCoA analysis revealed significant distinctions in microbiota composition between the MCAO and APG groups, illustrating that APG modified the intestinal flora of MCAO rats (Figure 3C). The APG group displayed a considerable increase in the abundance of Patescibacteria (Figure 3D). Moreover, we assessed the microbiota’s compositional structure at both the phylum and genus taxonomic levels through multi-sample composition histograms (Figures 3E, F). At the genus level, there was a significant rise in Parasutterella, Anaerostipes, the Christensenellaceae R-7 group, and Candidatus_Saccharimonas in the APG group, whereas Bifidobacterium, Akkermansia, and Rikenellaceae RC9 gut groups were significantly diminished in the APG group (Figure 3G). Additionally, we utilized PICRUSt2 analysis to forecast functional modifications in the intestinal microbiota of IS rats. Predictions derived from the KEGG database indicated that superpathways of glycolysis and mycothiol biosynthesis, among others, were markedly reduced in the APG group (*P* < 0.05) (Figure 3H).

**FIGURE 4:**
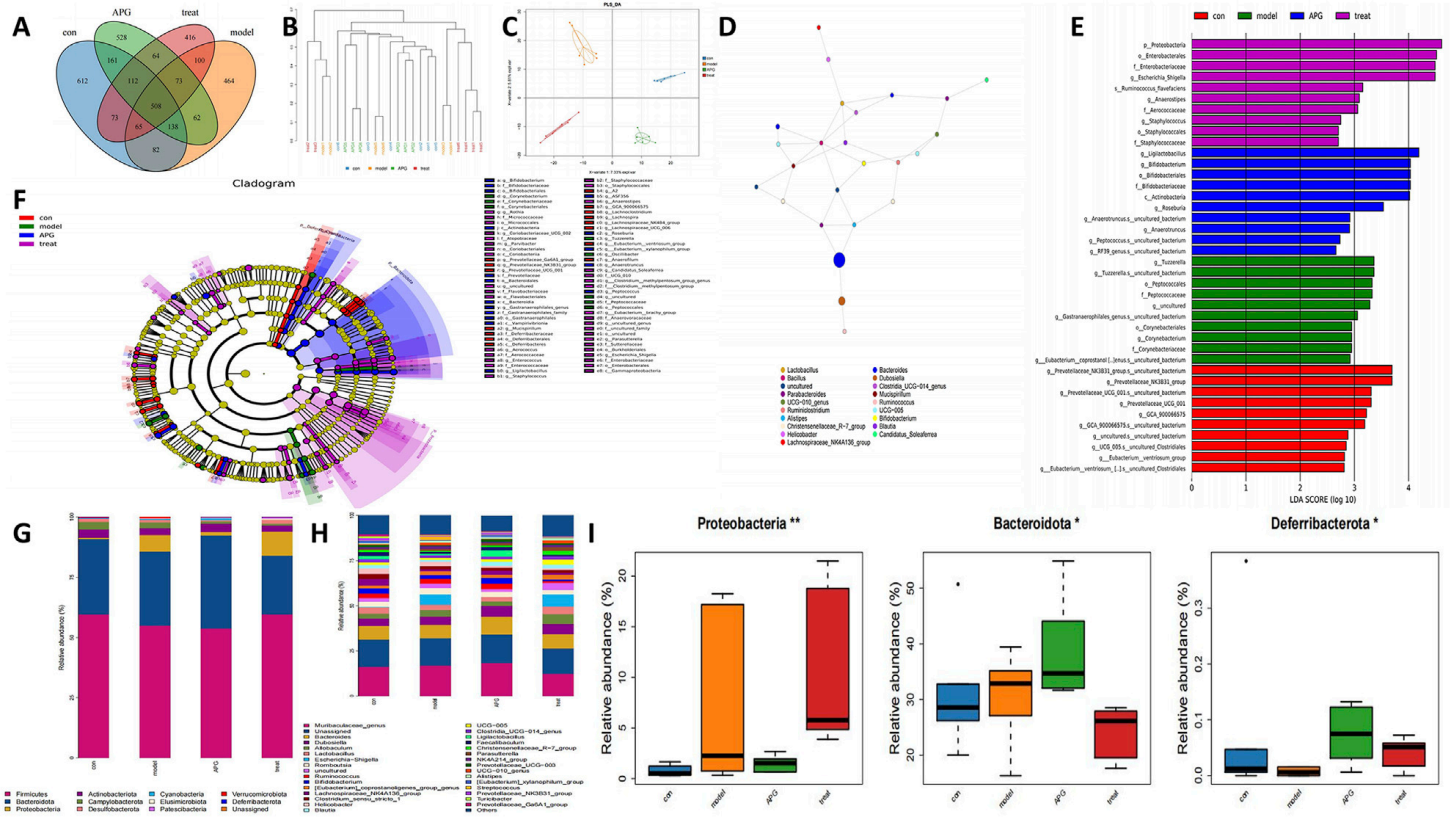
Gut microbiota changes in APG, MCAO, MCAO + APG compared with control group. **(A)** Venn diagram showing the number of OTUs in different groups. **(B)** The tree of hierarchical clustering. Different sets of samples are colored differently, and the length of the branches shows the distance between samples. The closer proximity of the samples implies a comparable makeup. **(C)** The bacterial community’s PCoA. The closer the distance, the greater the likeness. **(D)** Analysis of dominant species network diagrams. **(E)** Relative abundance of bacterial taxa is calculated by LEfSe tool. Each bar represents the log 10 effect size (LDA score) for the specific taxon. **(F)** Differences in microbiota across groups were analyzed by linear discriminant analysis (LDA) effect size (LEfSe) was analyzed. **(G)** The distribution of gut microbiota relative abundance at the phylum level. **(H)** The distribution of gut microbiota relative abundance at the genus level. **(I)** Bacterial communities with statistically significant differences in abundance at the phylum level. *p < 0.05, **p < 0.01, ***p < 0.001.

To examine the regulatory influences of control, MCAO, APG, and MCAO + APG rats, we collected fecal samples from various groups of rats and conducted 16S rRNA sequencing. The analysis revealed 612, 464, 528, and 416 unique OUTs across the control, MCAO, APG, and MCAO + APG groups, respectively. Overall, the combined total of OUTs from all four groups was 508 (Figure 4A). The cluster analysis indicated a significant difference in the composition of fecal species between the rats in the MCAO group and those in the MCAO + APG group. Similarly, notable differences were observed between the control and APG groups (Figure 4B). Principal Coordinate Analysis (PCoA) further demonstrated distinct differences in microbiota composition between the MCAO and MCAO + APG groups, and also between the control and APG groups. The introduction of APG modified the intestinal microbiota of the MCAO rats (Figure 4C). The analysis of the dominant species network revealed a close relationship between *Bacteroides*, *Lactobacillus*, and Alistipes (Figure 4D). Variations in microbiota among the groups were evaluated using Linear Discriminant Analysis Effect Size (LEfSe) (Figures 4E, F). Additionally, we explored the compositional structure of the microbiota at both the phylum and genus levels through multi-sample species composition histograms. The findings indicated that, at the phylum level, Bacteroidota showed a significant increase in the APG group compared to the control group, whereas the abundance of Firmicutes declined in the APG group. Interestingly, this trend was reversed in the APG + MCAO group when compared to the MCAO group (Figures 4G, H). The levels of Proteobacteria and Deferribacterota were significantly elevated when comparing the control with the APG group as well as the MCAO with the MCAO + APG group, while Bacteroidota exhibited the opposite trend (Figure 4I).

### 3.4 Metabolomics sequencing revealed changes in metabolites of MCAO after APG treatment

**FIGURE 5:**
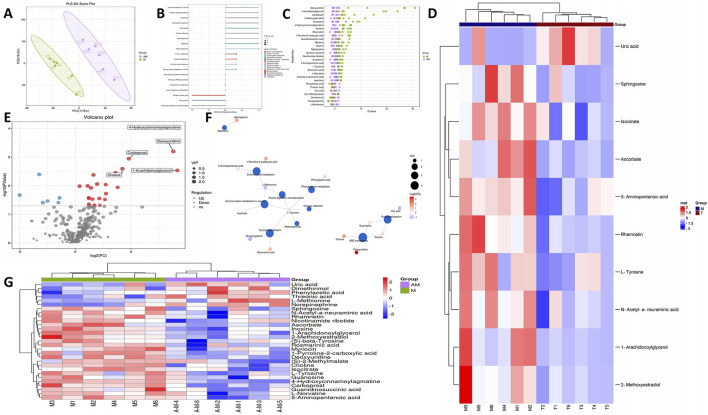
Alterations in the metabolites of MCAO and APG treatment rats. **(A)** PLS-DA score plot of APG vs MCAO. **(B)** KEGG of metabolic pathway enrichment. **(C)** Z-score plot. The vertical coordinate is the name of the metabolite, the color of the dots represents different groups, and the horizontal coordinate is he value obtained by converting the Z-score of the metabolite in the group, the more to the right, the more the metabolite is in the group. The horizontal coordinate is the relative content of the metabolite in the group converted by Z-score; the more to the right means the more metabolite in the group.t. **(D)** Top 10 metabolite heat map. **(E)** The volcano Plot indicates upregulated (red) and downregulated (blue) metabolites. **(F)** Analysis of dominant metabolite network diagrams. **(G)** Differential metabolite heat map.

We examined alterations in metabolites from rat brain samples through non-targeted LC-MS. Figure 5A illustrates that the metabolites exhibited significant variations between the control group and the model group. Furthermore, this study utilized the PLS-DA model for more comprehensive analysis. The PLS-DA score plots indicated distinctions between MCAO and APG + MCAO. KEGG analysis revealed that the primary pathways implicated included the AMPK signaling pathway, Glycolysis/Gluconeogenesis, and the Citrate cycle (TCA cycle), among others (Figure 5B; Supplementary Table S1). Figures 5C, D highlight that metabolites such as Ascorbate, L-Tyrosine, Sphingosine, N-Acetyl-a-neuraminic acid, Rhamnetin, 1-Arachidonoylglycerol, 5-Aminopentanoic acid, Uric acid, Isocitrate, and 2-Methoxyestradiol showed significant alterations in the model group. The volcanic map illustrating differential metabolites is presented in Figure 5E. D-Amino acid metabolism, Protein digestion and absorption, Tyrosine metabolism, and ABC transporter pathways were the most prevalent in the metabolite network (Figure 5F). A heat map of differential metabolites is shown in Figure 5G.

### 3.5 APG improvement IS affects ATP levels by improving the flora and metabolites

**FIGURE 6:**
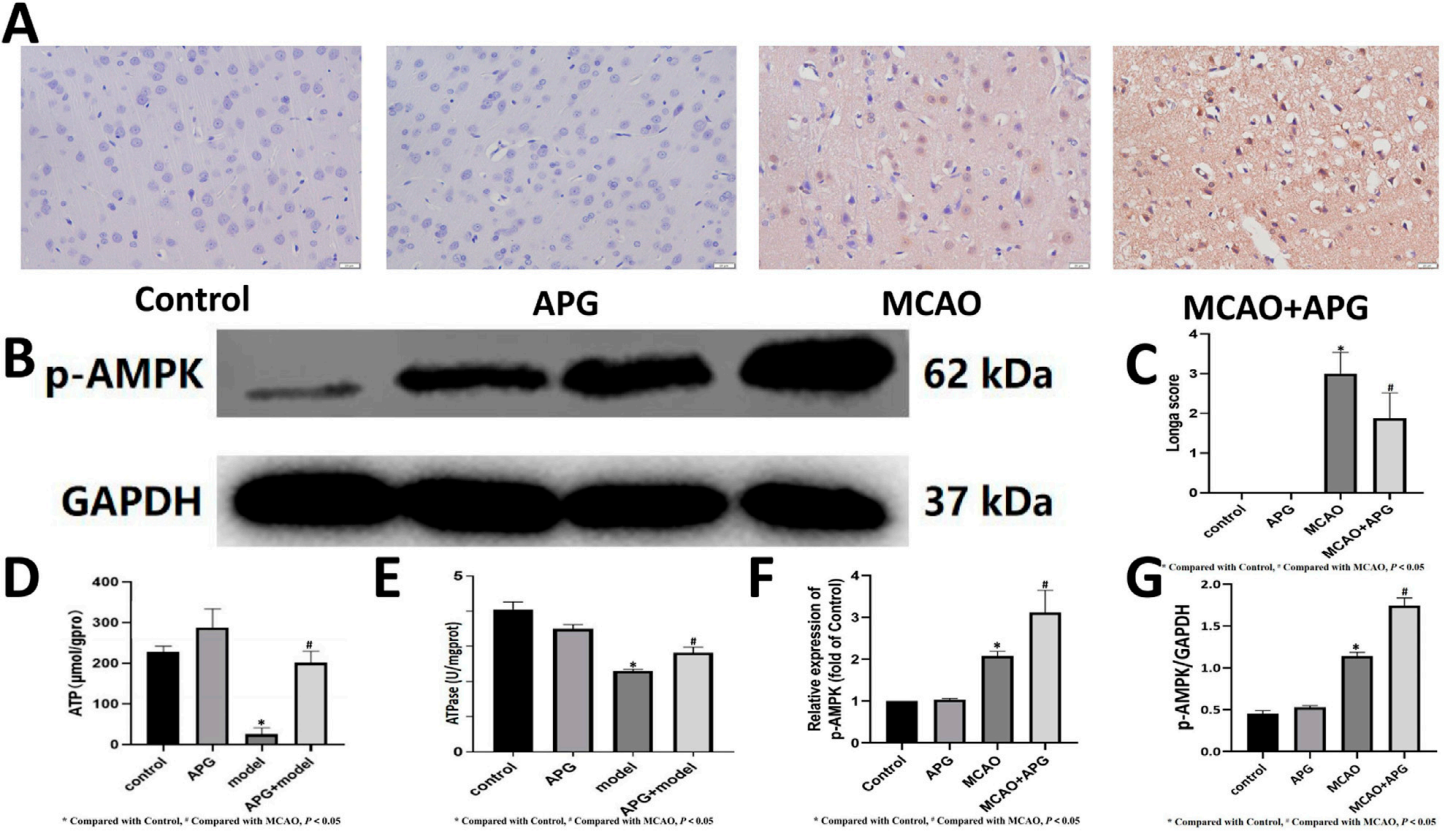
Effect of APG on energy metabolism in MCAO. **(A, F)**: Immunostaining photomicrographs of p-AMPK and quantitative analysis of the integrated optical density. Bar = 50 µm. **(B, G)**: Representative Western blot image of p-AMPK. The relative band densities of p-AMPK normalized with GAPDH, and data are expressed as mean ± SD (n = 3). *P < 0.05, compared with control; ^#^ *P* < 0.05, compared with MCAO. **(C)** Statistical graph of neurological deficit score. Data are expressed as mean ± SD (n = 8). **(D, E)**: Determination of ATP and ATPase content. Data are expressed as mean ± SD (n = 3). *P < 0.05, compared with control; ^#^ *P* < 0.05, compared with MCAO.

An analysis using immunohistochemistry and Western blot to assess p-AMPK staining was conducted to evaluate the energy metabolism in neurons located within the ischemic semi-dark band. The findings from this analysis indicated that AMPK levels were elevated in the APG group when compared to the MCAO group (refer to Figures 6A, B, F, G, *P* < 0.05). Furthermore, the levels of ATP and ATPase were lower in the MCAO group relative to the control group. Conversely, the ATP and ATPase levels in the MCAO + APG group exceeded those found in the MCAO group. Interestingly, although APG treatment led to an increase in ATP levels and a decrease in ATPase levels in comparison to the control group, these changes did not reach statistical significance (Figures 6D, E, P < 0.05).

### 3.6 APG improves inflammation and oxidative stress in ischemic stroke

The levels of MDA, IL1β, and IL6 were markedly increased, while the activities of SOD and GSH were notably decreased in the ischemic penumbra tissue of the MCAO group in contrast to the control group (*P* < 0.05). However, these alterations were mitigated in the groups treated with APG (*P* < 0.05) (Figures 1E–I).

## 4 Discussion

IS is a neurological condition characterized by the interruption of blood supply to the brain, leading to hypoxia and ischemic necrosis of brain tissue. Recent studies have revealed a significant relationship between the pathogenesis of IS and intestinal flora, which serves as an important microecological system in the human body (Peh et al., 2022). Intestinal flora, as the largest microecological community in the human body, possesses a complex composition and function that significantly impacts human health. This microbiota is not only involved in the digestion and absorption of nutrients but is also closely linked to the body’s immune system, metabolism, and nervous system. In the context of IS, increasing evidence suggests that intestinal flora may play a role in its pathogenesis (Zhu et al., 2016). On one hand, an imbalance in intestinal flora may promote the formation and progression of atherosclerosis by influencing lipid metabolism, inflammatory responses, and other mechanisms, thereby increasing the risk of IS. On the other hand, intestinal flora following an IS may also directly impact brain function by producing specific neurotransmitters or metabolites, which could influence the pathology of IS. APG has demonstrated significant efficacy in the context of IS. However, it remains unclear whether APG improves intestinal flora and metabolite profiles after an IS. Therefore, a thorough investigation into the relationship between APG’s regulation of intestinal flora and brain metabolism in IS is essential for enhancing our understanding of the mechanisms by which APG contributes to the treatment of IS.

We found that APG had a protective effect on neurons and nerve cells in IS. The Nissl body appears as a triangular or elliptical block of substance found within the cytoplasm of nerve cells. It can be stained purple-blue using a variety of dyes, including methyl violet, methylene blue, toluidine blue, and cresyl violet. The Nissl Stain Solution (using the toluidine blue method) exhibits several notable features: ease of use, consistent staining results, and broad applicability. This method can be utilized on paraffin tissue sections that include Nissl substances, Nissl bodies, neurons, and more. The presence and absence of Nissl bodies serve as critical indicators regarding nerve cell integrity. In cases of encephalitis, cerebral ischemia, or axonal reactions, Nissl bodies may dissolve or completely vanish. Research findings indicated that in the control group and APG-treated rats, the cortical structure was distinctly observable, showcasing large, round nuclei and an abundance of blue-stained Nissl bodies evident within the cell paddles. Conversely, in the MCAO model group, nerve fibers appeared disorganized, nuclei adopted triangular or spindle shapes, Nissl bodies dissolved or entirely disappeared, and their numbers were significantly diminished. Following treatment with APG in the MCAO model rats, there was a noticeable recovery of Nissl bodies, along with an increase in their quantity. HE staining revealed the presence of edema, structural vacuolization, nuclear condensation, and an elevation in eosinophils within the ischemic semi-dark band of rats in the MCAO group, which demonstrated significant improvement following treatment with APG.

Our intestinal microbiota results indicated that the level of Firmicutes in the MCAO group was reduced, while recovery following APG treatment was comparable to that of the control group. Firmicutes represent one of the most significant bacterial groups within organisms, playing a crucial role in host nutrition and metabolism through the synthesis of short-chain fatty acids, and contributing to the protective function of the intestinal barrier (Huang et al., 2018). Furthermore, Christensenellaceae_R-7 was found to be decreased in the MCAO group but exhibited a reversal after APG treatment. Christensenellaceae_R-7 is a prevalent class of microorganisms within the intestine, primarily distributed in regions such as the colon and cecum, and is essential for maintaining the intestinal microecological balance (Fournier et al., 2023). Christensenellaceae_R-7 is a bacterial group known for its ability to produce short-chain fatty acids, primarily propionic acid and butyric acid. These short-chain fatty acids play significant nutritional roles in the intestine, including providing energy for intestinal epithelial cells, promoting cell repair and regeneration, and inhibiting the growth of harmful bacteria, which collectively contribute to maintaining intestinal health. Additionally, Christensenellaceae_R-7 has been shown to positively influence blood pressure, which is a critical factor in the risk of IS(Calderón-Pérez et al., 2020). Among patients with lung cancer, the adverse reactions and survival time of the high Christensenellaceae_R-7 group were longer than those of the low Christensenellaceae_R-7 group (Chen et al., 2023). Additionally, Christensenellaceae_R-7 possesses characteristics that regulate immune system function. By influencing the integrity of the intestinal mucosal barrier, it modulates the activity of intestinal immune cells and enhances the body’s immune response. This modulation aids in the prevention and treatment of conditions such as intestinal inflammation and infections.

Blautia levels decreased in the MCAO group but increased following APG treatment. Blautia is an anaerobic microorganism that is commonly found in the intestines and feces of mammals, belonging to the phylum Firmicutes and the family Lachnospiraceae (Zhou et al., 2021). This microorganism possesses potential probiotic properties that can alleviate inflammation, promote the production of short-chain fatty acids (SCFAs), and maintain intestinal homeostasis (Mao et al., 2023). Notably, fecal Blautia levels in patients with IS are negatively correlated with the National Institutes of Health Stroke Scale (NIHSS) scores (Bao et al., 2024). Consequently, the increase in Blautia levels after IS due to APG treatment may help mitigate energy deficiency, inflammation, and damage to the intestinal barrier.

*Lactobacillus* levels decreased following MCAO and subsequently increased after APG treatment. As a member of the phylum Firmicutes, *Lactobacillus* is recognized as a key probiotic within the intestinal microbiota. It plays a crucial role in communicating with the intestinal epithelial lining to maintain the integrity of the intestinal barrier, inhibit the growth of pathogenic bacteria, and enhance the balance of the intestinal flora (Rahman et al., 2024). Furthermore, *Lactobacillus* exhibits protective effects against IS(Rahman et al., 2024). *Lactobacillus* has antibacterial activity, which can inhibit pro-inflammatory mediators and relieve intestinal inflammation (Rastogi and Singh, 2022). *Lactobacillus* has the ability to enhance both innate and acquired immunity, improve the body’s resistance to disease, and alleviate intestinal inflammation and infection (Huang et al., 2017). Constipation is a common symptom of IS and is associated with adverse outcomes (Su et al., 2009). *Lactobacillus* can also improve constipation and may have a potential protective effect against IS (Wang et al., 2023).

In the MCAO group, Oscillibacter levels decreased, while they increased following APG treatment. Oscillibacter is known to play a variety of important roles in organisms and contributes to the maintenance of intestinal flora balance. Notably, Oscillibacter levels are reduced in patients who have experienced an IS (Yin et al., 2015). Oscillibacter is also capable of producing short-chain fatty acids (SCFAs), including butyrate, which are crucial for maintaining intestinal health. These SCFAs can regulate intestinal pH and promote the growth and repair of intestinal cells (Yao et al., 2020). The gut microbiota significantly influences the development and function of the immune system. Oscillibacter, a component of the intestinal microbiota, plays a crucial role in regulating immune responses. It has been shown to promote immune cell function and enhance host resistance to pathogens. Additionally, Oscillibacter can modulate inflammatory responses, thereby preventing damage to the host caused by excessive inflammation (Bajaj et al., 2024). Oscillibacter has the potential to alleviate constipation and may also enhance outcomes in individuals with inflammatory syndromes (Kim et al., 2021). In summary, Oscillibacter plays several crucial roles in organisms, including the maintenance of intestinal health, participation in energy metabolism, regulation of the immune system, and its association with various diseases.

The levels of *Streptococcus* increased significantly in the IS group, while they decreased significantly in the APG group. *Streptococcus* is a potentially pathogenic bacterium, and its levels tend to rise following an IS (Zheng et al., 2022). *Streptococcus* can lead to various respiratory diseases, including pneumonia, otitis media, and sinusitis. Additionally, there is a notable association between *Streptococcus* and stroke. In addition, *Streptococcus* can cause intestinal barrier disruption (Wang et al., 2018).

In the MCAO group, the abundance of Prevotellaceae decreased; however, it increased following APG treatment. As a significant member of the intestinal microbiota, Prevotellaceae bacteria interact closely with the host’s immune system and may play a role in regulating immune responses. A study has found that a high abundance of Prevotellaceae in the gut is associated with a reduced risk of immune-mediated brain disease (Jeffery et al., 2017). Prevotellaceae levels were found to be inversely associated with stroke (Wang H. D. et al., 2022).

Recent studies have demonstrated a significant relationship between intestinal flora-mediated oxidative stress and various diseases. This process is regulated by the immune response mediated by intestinal flora, DNA damage, and intestinal inflammation (Sun et al., 2024). Our previous research indicated that DNA damage increases and oxidative stress surges following IS. PARP1 expression and DNA repair increased after APG treatment (Ping et al., 2024). Consequently, this study investigates the effects of APG on intestinal flora and metabolites in the aftermath of ischemic stroke. NAD+ serves as a substrate for PARP1, which necessitates its consumption during DNA repair, a process essential for maintaining genomic stability (Mendelsohn and Larrick, 2017). Furthermore, NAD+ interacts with glutathione by preserving the reduction state of GSH through the generation of NADPH. GSH is capable of eliminating free radicals, protecting NAD+-related metabolic enzymes, and ensuring a consistent supply of NAD+(Ghosh et al., 2014). Our study indicates that the increase in GSH content in Nicotinamide ribotide following APG treatment is closely associated with alterations in the intestinal microbiota (Ping et al., 2024).

We used brain metabolomics to obtain the effects of APG and its microbiota on brain metabolites in IS. Our metabolomics results found that the levels of Riboflavin, L-Tryptophan, Pyridoxine, etc., were decreased in the APG group. Atherosclerosis is both a risk factor and a causative agent of ischemic stroke. The gut bacterium *Bacteroides* caccae has been shown to promote atherothrombosis by regulating L-tryptophan metabolism (Liu et al., 2024). Pyridoxine levels are typically diminished due to alterations in intestinal flora following various diseases (Xia et al., 2024). Pyridoxine is closely associated with oxidative stress within the glutathione peroxidase system (Dalto and Matte, 2017). On the contrary, the contents of Nicotinamide ribotide, GSH, Uric acid, and AMP were increased in the APG group. The reduction in vitamin B and L-Tryptophan levels in brain tissue following APG treatment may facilitate the production of Nicotinamide riboside, which can enhance energy supply and support DNA repair mechanisms (Wilk et al., 2020; Cuny et al., 2022; Tiwari et al., 2022). Intestinal flora is closely linked to glutathione levels (Mardinoglu et al., 2015). Clinical studies have demonstrated that the abundance of Escherichia-Shigella in patients with hyperuricemia is significantly higher, while the abundance of Oscillospiraceae is significantly lower (Zhang et al., 2022). Consequently, APG may influence metabolite changes following ischemic stroke through modulation of intestinal flora.

In addition, the antioxidant markers glutathione and uric acid were increased after APG treatment. In addition, AMP levels were higher after APG treatment than in the MCAO group. AMP is involved in cellular energy metabolism after IS by AMPK (Wan et al., 2016). These results indicated that APG treatment and APG-improved microflora promoted cerebral oxidative stress and energy metabolism in IS. This is probably done through the AMPK signaling pathway.

Based on the results of the metabolome and microbiota analyses, we examined the effect of APG on energy metabolism in MCAO rats. Our findings indicate that both ATP content and ATPase levels in the MCAO group increased following APG treatment, suggesting an improvement in energy metabolism post-MCAO. Additionally, immunohistochemistry revealed a reduction in the expression of p-AMPK in the MCAO group, which was reversed by APG treatment. This suggests that APG may modulate energy metabolism after MCAO through the AMPK signaling pathway. AMPK plays a crucial role in IS as it is a key molecule in the regulation of bioenergetic metabolism. During IS, AMPK serves a significant protective function by maintaining the balance of brain energy metabolism, primarily through the enhancement of ATP anabolism and the inhibition of ATP decomposition (Zhu et al., 2019). In addition, APG improved oxidative stress and inflammation in IS penumbra brain tissue.

## 5 Conclusion

In summary, this study elucidates a novel mechanism through which APG safeguards IS by modulating intestinal flora and brain metabolism. Furthermore, the protective effect of APG on IS is mediated by the upregulation of AMPK expression. Consequently, APG holds promise as a potential therapeutic agent for IS. However, this study has limitations, including the lack of a rescue test and the omission of germ-free mice for fecal bacterial transplantation. These shortcomings will be addressed in future experiments. In addition, our experiment lacked female rats. Due to the protective effect of estrogen on IS and the difference of sex on intestinal microbiota in IS also need to be confirmed in future experiments. This research lays a theoretical foundation for the clinical application of APG.

## Data Availability

The raw data is uploaded to the MetaboLights database, accession number MTBLS12272.
